# Exploring the association between specific genes and the onset of idiopathic scoliosis: a systematic review

**DOI:** 10.1186/s12920-022-01272-2

**Published:** 2022-05-19

**Authors:** Sergio De Salvatore, Laura Ruzzini, Umile Giuseppe Longo, Martina Marino, Alessandra Greco, Ilaria Piergentili, Pier Francesco Costici, Vincenzo Denaro

**Affiliations:** 1 Research Unit of Orthopaedic and Trauma Surgery, Fondazione Policlinico Universitario Campus Bio-Medico, Via Alvaro del Portillo, 200, 00128 Rome, Italy; 2 Research Unit of Orthopaedic and Trauma Surgery, Department of Medicine and Surgery, Università Campus Bio-Medico Di Roma, Via Alvaro del Portillo, 21, 00128 Rome, Italy; 3 Department of Orthopedics, Children’s Hospital Bambino Gesù, 00165 Palidoro, Rome, Italy

**Keywords:** Scoliosis, Idiopathic scoliosis, Early onset, Genetic, Diagnosis

## Abstract

**Background:**

Idiopathic Scoliosis (IS) is the most common spinal deformity in adolescents, accounting for 80% of all spinal deformities. However, the etiology remains uncertain in most cases, being identified as Adolescent Idiopathic Scoliosis (AIS). IS treatments range from observation and sport to bracing or surgery. Several risk factors including sex and familiarity, have been linked with IS. Although there are still many uncertainties regarding the cause of this pathology, several studies report a greater incidence of the defect in families in which at least one other first degree relative is affected. This study systematically reviews the available literature to identify the most significant genes or variants related to the development and onset of IS.

**Methods:**

The research question was formulated using a PIOS approach on the following databases: Medline, Embase, Cinahl, Scopus, Web of Science and Google Scholar. The search was performed from July to August 2021, and articles from the inception of the database to August 2021 were searched.

**Results:**

24 of the 919 initially identified studies were included in the present review. The 24 included studies observed a total of 16,316 cases and 81,567 controls. All the considered studies stated either the affected gene and/or specific SNPs. CHD7, SH2B1, ESR, CALM1, LBX1, MATN1, CHL1, FBN1 and FBN2 genes were associated with IS development.

**Conclusions:**

Although association can be found in some candidate genes the field of research regarding genetic association with the onset of IS still requires more information.

## Background

Idiopathic Scoliosis (IS) is the most common spinal deformity in adolescents, accounting for 80% of all spinal deformities. However, the etiology remains uncertain in most cases, being identified as Adolescent Idiopathic Scoliosis (AIS) [1, 2]. Diagnosis of IS begins with a complete physical examination that starts with inspecting shoulder and flank asymmetry. Clinical evaluation is of fundamental importance for the efficacy of the treatment [3]. According to the Scoliosis Research Society classification, scoliosis could be divided into early (EOS) or late-onset; the latter is usually identified with AIS. EOS is characterized by its appearance in children before ten years [4, 5]. It is a complex and highly variable condition, with several etiologies, manifestations, and associations [6]. EOS accounts for less than 1% of the total scoliotic cases and, several conditions including genetic syndromes and neurological diseases, could explain its onset [3, 6]. Among these conditions, VACTERL syndrome is notably associated with congenital scoliosis. Other pathologies also appear to be related to the onset of EOS, in particular neuromuscular disorders ( syringomyelia or myelomeningocele), connective tissue disorders (Marfan Syndrome) and metabolic conditions (osteogenesis imperfecta) [3]. AIS presents in patients older than 10 years of age with a global incidence of 3% [7]. Despite the high incidence of cases worldwide, AIS etiology remains unclear [8]. IS treatments range from observation and sport, to bracing or surgery [1, 3, 9]. In the latter approach the procedure aims to stop curvature progression before reaching a severe spinal curvature identified when the Cobb Angle is greater than 90° and that could reduce cardio-pulmonary function. Bracing is another procedure which aims to achieve halting or reduction of curvature progression but acts using external compressive forces [10]. Despite being a non-invasive approach, contrarily to surgery, bracing is not free from side effects, as it has proven to produce a reduced lung volume accompanied by increased effort during breathing [10].

Several risk factors such as sex and familiarity, have been linked with IS [7]. Moreover, variation in the distribution of the disease in different countries has been reported [11]. However, the precise etiology of this condition remains unknown, and no clear genetic or environmental factors have been directly associated with IS. Although there are still many uncertainties regarding the cause of this pathology, several studies report a greater incidence of the defect in families in which at least one other first degree relative is affected; this information has been supported by twin studies [7, 12]. According to these studies, it is possible to hypothesize that there may be a relevant genetic contribution to the development of IS [13].

IS management is strictly related to the time of presentation and the value of the Cobb angle. The study by Weinstein et al. indicated bracing as an effective AIS treatment option in the case of non-surgical scoliosis (< 45° of Cobb angle). Another study by Hans-Rudolf Weiss reported that patients not treated for IS in the early stages of the disease (skeletal maturity and > 45°) tended to have worse outcomes compared to ones treated early [14].

Therefore, an early diagnosis and treatment could reduce the risks of intervention; furthermore, these improvements could lead to a decrease in the overall rate of complications in case of surgery.

Genetic tests could diagnose IS before the beginning of characteristic symptoms, allowing early diagnosis and treatment. To our knowledge, however, few studies investigated specific genes related to IS onset. In the light of these considerations, the importance of refining strategies to predict and prevent the disease is evident and may be crucial to diagnosis and treatment.

This study systematically reviews the available literature to identify the most significant genes or variants related to the development and onset of IS.

## Methods

### Study Selection

The research question was formulated using a PIOS-approach: Patient (P); Intervention (I); Outcome (O) and Study Design (S). This systematic review aims to study the association (O) between patients that have developed IS (P) and specific genes, identified through genetic screening. Literature in which patients affected with IS were genetically tested (I) for mutations in genes of interest was reviewed. The following study designs were included (S): Randomized Controlled Trials (RCT) and Non-Randomized (NRCT) as Prospective (PS), Retrospective (RS), Case series (CS), Case–Control (CC), and Cohort (CS) studies.

### Inclusion Criteria

Only articles published in English were screened. Peer-reviewed articles of each level of evidence according to Oxford classification were considered. Only studies reported on affected genes in the onset of IS in patients were included.

### Exclusion Criteria

Technical notes, letters to editors, instructional courses or studies that did not include genetic testing of patients were excluded. Studies with a sample size smaller than 10 patients were considered not eligible for the present study. Studies with missing or incomplete data were also excluded. The analysis did not include degenerative, syndromic, and neurological scoliosis.

### Search

A systematic review was performed using the Preferred Reporting Items for Systematic Reviews and Meta-analyses (PRISMA) guidelines. Medline, EMBASE, Scopus, CINAHL and CENTRAL bibliographic databases were searched using the following string: ((diagnosis) AND ((genetic) OR (genome))) AND ((scoliosis) AND ((((adolescent) OR (idiopathic)) OR (early-onset)) OR (late-onset))). Keywords were used both isolated and combined. Additional studies were searched among reference lists of selected papers and systematic reviews.

The search was performed by two authors (A.G. and M.M.) from July to August 2021 and articles from the inception of the database to August 2021 were searched.

### Data Collection Process

**Fig. 1:**
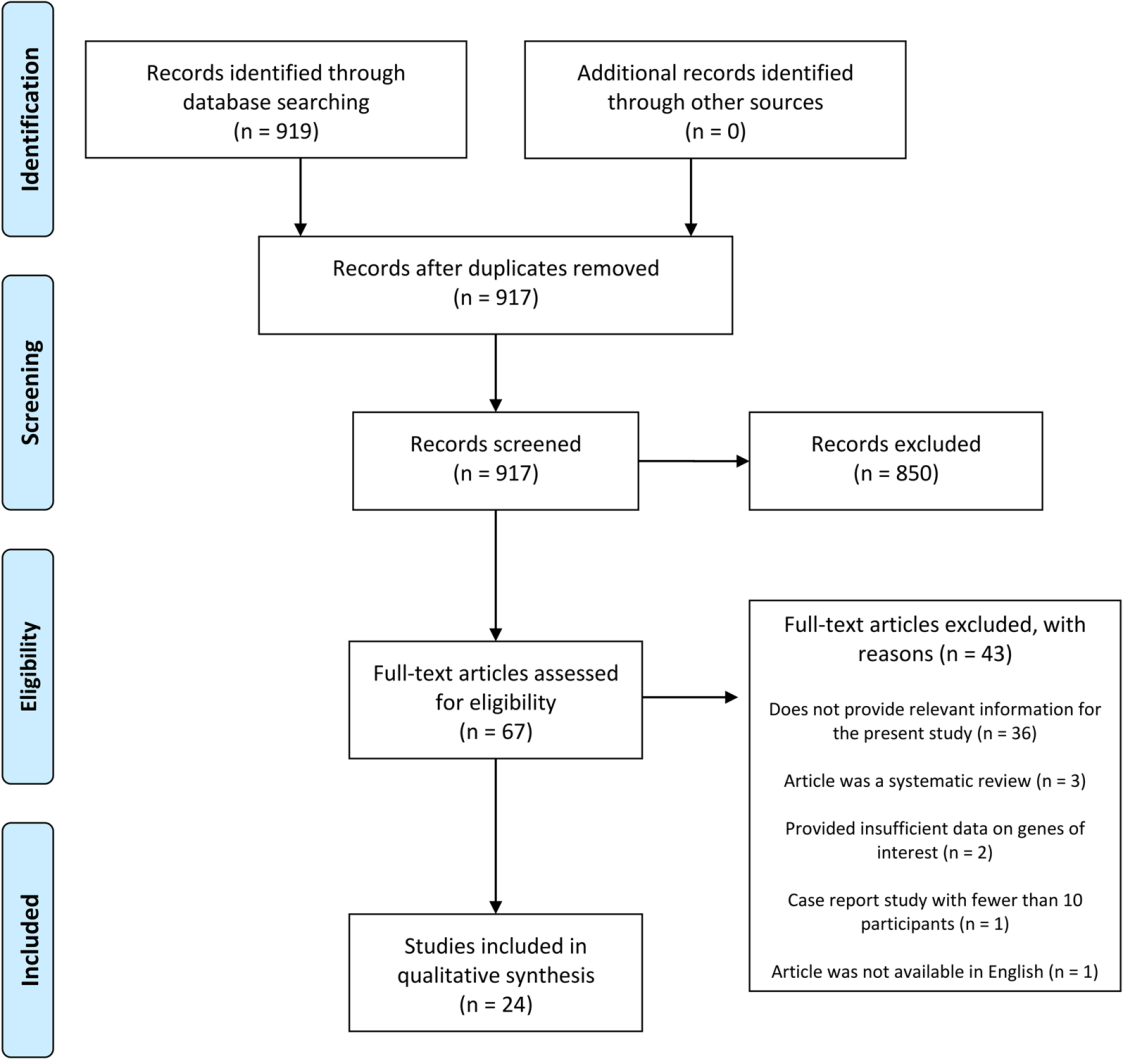
PRISMA Flowchart

Two independent reviewers performed data collection (A.G. and M.M.), and differences were reconciled by mutual agreement. Any disagreement was resolved upon consultation of a third reviewer (S.D.S.). Firstly, title and abstract screening were performed, and then selected texts were reviewed in full text. The PRISMA flowchart, seen in Fig. 1, reported the inclusion and exclusion of reviewed articles.

### Data Items

General study characteristics extracted included: primary author, year of publication, country, type of study, level of evidence, sample size (cases and controls), affected gene, statistical association (expressed by p-value or odds ratio), diagnostic method, type of scoliosis (early or late-onset).

### Risk of Bias

The non-randomized control studies included in this review were assessed for the possibility of bias using the Risk of Bias in Non-Randomized Studies of Interventions (ROBINS-I) tool by Cochrane. Cochrane's Risk of Bias 2 (RoB 2) tool was used to test for bias in randomized control studies. The scoring was performed by the authors A.G. and M.M. independently, and any disagreement was resolved by a third author S.D.S.

## Results

### Study Selection

The search resulted in 919 records identified, which went down to 917 after duplicate removal. Of the 917 records, 850 were excluded during title/abstract screening, leaving 67 articles for the full-text assessment. After the full-text assessment, 43 articles were not considered eligible for the study: some did not provide relevant information for the present review (n = 36) or provided insufficient data on the genes of interest (n = 2); one study was excluded because it included less than 10 participants and one article was not available in English. Thus, 24 studies were included for qualitative synthesis. Due to different identified genes in the collected data, a meta-analysis could not be performed.

### Study Characteristics

The 24 included studies observed a total of 16,316 cases and 81,567 controls. The high number of controls compared to cases is mostly accounted by Kou et al., who utilized three large genome wide association studies (GWAS) that included 73,884 controls compared to 5,327 cases.

All the considered studies stated either the affected gene and/or specific single nucleotide polymorphisms (SNPs). Of the 23 studies 15 stated SNPs of the affected gene[2, 8, 12, 15–26], seven of these also presented the risk allele and/or genotype[15, 16, 18, 21, 23, 25, 26]. Of those that didn’t report the related SNPs, three specified affected gene locus only[1, 7, 27], one stated the specific variant of the affected gene[28], two specified affected gene locus and deletion or insertion, and/or risk allele[4, 29], one stated copy number variant (CNV) of affected gene and corresponding duplication or deletion[9], and one stated risk allele genotype of the affected gene[30].

In regards to statistical data extracted, 11 studies included only the p-value[1, 2, 7, 9, 18–20, 24–26, 28], 11 included both p-value and odds ratio[8, 12, 15–17, 21–23, 27, 30, 31], and two provided neither [4, 29]. In the case of absent p-value and/or odds ratio the item was not assessed, and the studies were used to identify other possible genes of interest.

Of the selected studies, the following levels of evidence and study designs were included: 10 level III case–control studies[2, 16, 19, 20, 23–25, 27, 29, 30], seven level III cohort studies[1, 4, 7, 8, 15, 17, 21], three level III case–control cohort studies[9, 12, 31], three level III case–control association studies[18, 22, 26], one level IV cross-sectional study[28]. The study characteristics are reported in Table 1.

**Table 1:** Primary author, year of publication, country, type of study, level of evidence (LOE), diagnosis method, age of IS on-set and associated pathology of the included studies

Author, year	Country	Type of study, level of evidence	DNA extraction protocol	Age of IS onset		Associated pathology
				Early Onset	Late Onset	
Alden, 2006	USA	Cohort study, Level 3	Standard Purification Protocols			
Borysiak, 2020	Poland	Cohort study, Level 3	AxyPrep Blood Genomic DNA Miniprep Kit			
Buchan, July 2014	USA	Case–control cohort study, Level 3	Isolation Kit for Mammalian Blood or Oragene1 Purifier		x	Trisomy X, 1.8% (2/114)
Buchan, May 2014	USA, China	Case–control cohort study, Level 3	Genome Analyzer IIx or HiSeq 2000 sequencer SureSelect Human All Exon 38 Mb and 50 Mb kits or TruSeq Exome Enrichment kit		x	Marfan Syndrome
Kotwicki, 2014	Poland	Case–control study, Level 3	PCR restriction fragment length polymorphism (PCR–RFLP)			
Kou, 2019	Japan	Cohort study, Level 3		x		
Liu, 2017	China	Case–control association study, Level 3	Sequenom MassARRAY SNP genotyping platform			
Moon, 2013	South Korea	Case–control study, level 3	Single base primer extension assay		x	
Nikolova, 2016	Bulgaria, Japan	Case–control study, Level 3	PCR	1–3 years of age (4) 3–9 years of age (23),	10–16 years of age (78)	
Ogura, 2013	Japan	Retrospective cohort study, Level 3	Invaer Assay			
Sadler, 2019	USA	Cohort study, Level 3	IDT xGen Exome Panel V1 capture on Illumina HiSeq 4000 paired-end reads	x	x	
Sharma, 2011	USA	Case–control cohort study, Level 3	Genotyped on Illumina Human CNV370-Quad arrays			
Takahashi, 2018	Japan	Case–control study, Level 3	PCR-based Invader assay		x	
Takeda, 2017	Japan	Case–control study, Level 3	TaqMan real-time quantitative PCR, Microsatellite analysis, Sanger sequencing			Vertebral malformations
Wang, 2008	China	Cohort study, Level 3	PCR	x	x	
Wang, 2020	China	Cross-sectional study, Level 4	Whole-exome sequencing		x	
Wu, 2006	China	Case–control study, level 3	PCR, Electrophoresis			
Xu, 2015	China	Retrospective case control study, Level 3	TaqMan SNP Genotyping Assay		x	
Xu, 2020	China	Case–control association study, Level 3	Genome DNA Extraction with QIAGEN kit, Sanger Sequencing (10%), Exon Sequencing (192)			
Yilmaz, 2012	Turkey	Case–control study, level 3	RT-PCR			
Zhao, 2009	China	Case–control study, Level 3	QIAamp DNA Blood Mini kit		x	
Zhao, 2020	China	Cohort study, Level 3		x		
Zhou, 2012	China	Case–control study, Level 3	PCR restriction fragment length polymorphism (PCR–RFLP)		x	
Zhu, 2014	China	Case–control association study, Level 3				

*PCR* Polymerase Chain Reaction, *Dup* Duplication, *Del* Deletion, *RT-PCR* real-time polymerase chain reaction

### Gene and allele association

All data discussed in the following section are reported in Table 2.

**Table 2:** Primary author, year of publication, affected gene, frequency in cases and statistical association of the included studies

Author, year	Affected gene	Sample size		Frequency in cases		Statistical assocation	
		Cases	Controls	Cases	Controls	P value	Odds ratio
Alden, 2006	Chromosome 19p13: D19S591 D19S1034 D19S922 D19S714	703	495	Not Reported	Not Reported	0.0233* 0.0366* 0.0018(singlepoint)* 0.042 (multipoint)* 0.035*	
Borysiak, 2020	Gene: CHD7 rs1017861 G: A: GG: GA: AA: rs4738824 G: A: GG: GA: AA: rs4738813 T: C: TT: CT: CC:	211	83	(%) rs1017861 87.7 12.3 77.2 28 1.9 rs4738824 81.8 18.3 65.9 31.7 2.4 rs4738813 68.7 31.2 48.2 41 10.8	(%) rs1017861 74.6 25.4 55.4 38.6 6.0 rs4738824 79.5 20.5 63.9 32.1 4.8 rs4738813 69.3 30.7 49.4 39.8 10.8	rs1017861 Alleles: 0.0001 Dominant Model: 0.06* Recessive Model: 0.002* rs4738824 Alleles: 0.53 Dominant Model: 0.47 Recessive Model: 0.84 rs4738813 Alleles: 0.97 Dominant Model: 0.99 Recessive Model: 0.97	2.4 (1.5–3.8) 3.3 (0.9–12.7) 0.4 (0.2–0.6) 0.84 (0.6–1.2) 2.1 (0.6–7.9) 0.9 (0.5–1.6) 0.97 (0.66–1.44) 0.99 (0.44–2.25) 0.96 (0.58–1.59)
Buchan, July 2014	CNV: 16p11.2 1q21.1 duplication (proximal) 2q13 duplication 15q11.2 deletion 15q11.2 duplication 16p11.2 duplication	143	1079	(n) 3 1 1 1 1	(n) 1 7 4 5 2	0.0057* 0.6316 0.4639 0.5269 0.3118	
Buchan, May 2014	FBN1 FBN2 FBN1 or FBN2	323	493	(n) 13/311 11/316 24/304	(n) 5/489 5/427 10/425	0.0041* 0.0307* 0.000546*	4.2 3.0 3.5
Kotwicki, 2014	Gene ESR2 C/T rs1256120 A/G rs4986938 A/G rs1256049	248	243	Not Reported	Not Reported	0.1716	(0.2646–1.886) (0.6234–1.276) 1.557
Kou, 2019	LOC101928978: rs141903557 MTMR11: rs11205303 ARF1: rs12029076 TBX1: rs1978060 LINC02378/MIR3974: rs2467146 CSMD1: rs11787412 KIF24: rs188915802 BCKDHB/FAM46A: rs658839 CREB5: rs160335 NT5DC1: rs482012 LOC101927021/UNCX: rs11341092 PLXNA2: rs17011903 AGMO/MEOX2: rs397948882 FTO: rs12149832 LINC01514/LBX1: rs11190870 ADGRG6: rs9389985 BNC2: rs7028900 ABO: rs144131194 PAX1/LINC01432: rs6047716 CDH13: rs2194285	5327	73,884	Risk Allele Frequency 0.060 0.24 0.81 0.49 0.70 0.42 0.019 0.54 0.54 0.74 0.33 0.11 0.11 0.82 0.66 0.48 0.46 0.58 0.51 0.13	Risk Allele Frequency 0.047 0.21 0.78 0.47 0.67 0.38 0.013 0.51 0.51 0.72 0.31 0.10 0.10 0.79 0.56 0.43 0.42 0.55 0.47 0.11	9.78 × 10 − 11* 1.62 × 10 − 10* 2.17 × 10 − 10* 3.26 × 10 − 10* 5.96 × 10 − 10* 1.32 × 10 − 9* 1.94 × 10 − 9* 3.15 × 10 − 9* 9.10 × 10 − 9* 2.30 × 10 − 8* 2.92 × 10 − 8* 3.56 × 10 − 8* 3.66 × 10 − 8* 4.40 × 10 − 8* 2.01 × 10 − 82* 3.51 × 10 − 20* 2.19 × 10 − 17* 1.35 × 10 − 11* 1.45 × 10 − 11* 8.69 × 10 − 9*	1.33 1.17 1.18 1.16 1.15 1.14 1.66 1.14 1.13 1.14 1.14 1.20 1.20 1.16 1.52 1.21 1.20 1.15 1.15 1.19
Liu, 2017	Gene: LBX1 rs11190870 allele: C allele: T rs1322331 allele: T allele: G rs4917933 allele: A allele: G rs625039 allele: A allele: G rs11190872 allele: T allele: C	180	182	(n) 150 210 182 178 20 340 124 236 13 347	(n) 195 169 138 226 26 336 155 209 22 342	1.34 × 10–3* 6.15 × 10–4* 0.371 2.45 × 10–2* 0.127	
Moon, 2013	CHL1 rs10510181 DSCAM rs2222973 LAPTM4B rs2449539 FOXB1 rs1437480 CBLN4 rs448013 RRAGC rs10493083 BRIP1 rs16945692 MATN1 rs1149048 MTNR1B rs4753426 IGF1 rs5742612	35	68	Not Reported	Not Reported	(Allele) 0.965 0.207 0.002* 0.875 0.114 0.363 0.286 0.750 0.152 0.059	
Nikolova, 2016	Gene: IL-6 rs1800795	105	210	(%) (G = Risk Allele) GG: 51.4 CG: 38.1 CC: 10.5 G: 70.5%	(%) GG: 30.0 CG: 44.8 CC: 25.2 G: 52.4%	< 0.0001*	
Ogura, 2013	rs7613792 rs16902899 rs2700910 rs10787096 rs1558729 rs17635546	2117		Not Reported	Not Reported	0.66 1 1 0.701 1 1	0.84 (0.36–1.94) 0.99 (0.23–4.15 N/A 1.39 (0.31–6.24) N/A N/A
Sadler, 2019	Gene: SH2B1 1q21.1 2q13 15q11.2 15q13.3 16p13.11 Distal 16p11.2 Proximal16p11.2 HNPP/CMT1A 17q12 DiGeorge/VCFS	1197	1664	(n) Dup: 1 Del: 3 Del: 2, Dup: 0 Dup: 1 Del: 1, Dup: 1 Del: 0, Dup: 8 Del: 1 Del: 1 Del: 1, Dup: 1 Del: 2	(n) Dup: 0 Del: 0 Del: 2, Dup: 2 Dup: 0 Del: 2, Dup: 1 Del: 1, Dup: 1 Del: 0 Del: 1 Del: 0, Dup: 1 Del: 0	Dup: 0.42 Del: 0.07 Del:0.56, Dup:1 Dup: 0.42 Del:0.80, Dup:0.66 Del: 1, Dup: 0.005* Del: 0.42 Del: 0.66 Del: 0.42, Dup: 0.66 Del: 0.18	
Sharma, 2011	Gene: CHL1 rs1400180 rs9754850 rs9754552 rs10510181	375	444	$ 0.43 0.51 0.51 0.38	$ 0.41 0.44 0.44 0.30	0.56 0.044* 0.049* 0.021*	1.09 1.35 1.34 1.42
Takahashi, 2018	Gene: LBX1 rs11190870	2191		(n) (T = Risk Allele) TT: 818 TC: 865 CC: 177	Not Reported	0.13	
Takeda, 2017	Gene: TBX6 16p11.2del c.699G > A c.156delG c.935_936insGA c.333G > T	94		$$$ (n) 5 1 1 1 1	Not Reported		
Wang, 2008	Gene: TPH1 Allele A of rs10488682 A/Ahomozgote genotype	103	108	$$ (%) 19.9 39.8	(%) 7.9 15.7	0.0003* 0.001*	2.909
Wang, 2020	Missense variant in ESR1 (c.868A > G) Missense variant in ESR2 (c.236 T > C)	113		Not Reported	Not Reported	0.026* 0.014*	
Wu, 2006	PvuII, XbaI polymorphisms of Estrogen Receptor Gene PPXX PPXx PPxx PpXX PpXx Ppxx ppXX ppXx ppxx	174	202	(n), (%) 19, 9.40 8, 3.96 12, 5.94 21, 10.40 43, 21.29 28, 13.86 14, 6.93 25, 12.38 32, 15.84	(n), (%) 13, 7.47 13, 7.47 14, 8.05 8, 4.60 36, 20.69 26, 14.94 5, 2.87 17, 9.77 42, 24.14	0.53 0.139 0.422 0.036* 0.887 0.766 0.073 0.424 0.044*	1.29 0.51 0.72 2.41 1.04 0.92 2.52 1.30 0.59
Xu, 2015	allele G of rs12618119: allele A of rs9945359: allele T of rs4661748: allele C of rs4782809:	990	1188	$$ (%) 46.5 22.6 15.6 42.4	(%) 40 18.4 19.4 47.4	< 0.001*	1.29 1.29 0.77 0.82
Xu, 2020	Gene: SLC39A8 rs11097773	192	192	(G = Risk Allele) (n) GG: 2 AG: 26 AA: 164	(n) GG: 6 AG: 45 AA: 141	0.01*	0.486
Yilmaz, 2012	MCM6 (6p21) MATN-1 (1p35) VFR BsmI (12q13.1)	54	53	(n), (%) CC: 47, 89 CT: 6, 11 TT: 0, 0 AA: 20, 37.7 AG: 23, 43.3 GG: 10 (19%) GG: 19, 36 AG: 26, 49 AA: 8, 15	(n), (%) CC: 48, 88 CT: 5, 9.2 TT: 1, 1.8% AA: 16, 29.6 AG: 28, 51.8 GG: 10, 18.5 GG: 22, 40.74 AG: 26, 48.15 AA: 6, 11.11	0.97 0.66 0.59	1.16 (0.3–4.0) 1.17 (0.6–2.1) 0.8 ( 0.5–1.5)
Zhao, 2009	Gene: CALM1 rs12885713 rs5871 Gene: ER1 rs2234693	67	100	(n), (%) C allele—T allele 96 (71.6)—38 (28.4) 59 (44)—75 (56) 41 (30.6)—93 (69.4)	(n), (%) C allele—T allele 163 (81.5)—37 (18.5) 109 (54.5)—91 (45.5) 88 (44)—112 (56)	0.034* 0.061 0.014*	
Zhao, 2020	Gene: TBX6 16p11.2del	447		(n) 41	Not Reported		
Zhou, 2012	Gene: IL-17RC allele G of rs708567 GG genotype	529	512	$$ (%) 90.17 95.1	(%) 85.55 92.8	0.028* 0.023*	
Zhu, 2014	Gene: SOCS3 rs4969168 AA AG GG A G	398	367	(n) 56 215 127 327 469	(n) 49 208 110 306 428	AA: 0.587 A: 0.835	

^$^Case: Control risk allele frequencies; $$percentage of patients and controls with variant gene/deletion; $$$number of patients with variant gene/deletion; * p < 0–05

### Region 19p13

Alden et al. [1] evaluated four markers of region 19p13: D19S591, D19S1034, D19S922, and D19S714 with a statistically significant association (p < 0.005). Marker D19S1034 specifically has the strongest statistical association, suggesting that it may be more critical in the development of IS compared to the others.

### CNV 16p11.2

Four of the included studies reported on CNV 16p11.2, Buchan et al. reported on various deletions and duplications; however, the proximal duplication 1q21.1 was the only one that showed a significant correlation to the onset of IS given its p-value of 0.0057 [9].

Sadler et al. focused on gene SH2B1 with a 16p11.2 distal deletion and duplication [7], which seems to be the only alteration related to IS onset.

In two other studies, Takeda et al. and Zhao et al. identified a 16p11.2 deletion [4, 17, 29] in relation to the TBX6 gene, and both did not specify the statistical association.

### CHD7

Borysiak et al. focused on three SNPs of the CHD7 gene. However, only rs101786 demonstrates a statistical association [15]. These values suggest a strong association between the recessive model of rs101786 and IS development.

### TBX6

Kou et al. identified a specific SNP, of gene TBX6 rs1978060, with a statistically significant association between gene modification and IS onset [17].

### ESRs

Estrogen Receptor Genes (ESRs) may be related to IS, specifically ESR1 and ESR2. In the three tested SNPs, Wang et al. reported a significant association for the missense variant in ESR1 and another missense variant in ESR2 [16, 28]. Zhao et al. reported similar results ESR1 founding a significant relation to SNP rs2234693 [24] and IS. Wu et al. looked at PvuII and XbaI polymorphisms of the ESR gene and nine possible genotypes. They found that PpXX had a statistically significant correlation with the onset of IS [30]. However, in the study by Kotwicki et al., no association between IS and ESR2 was found. These data points hint at an association between estrogen receptor gene modifications and the onset of IS, despite Kotwiki et al. data showing no association.

SNP rs12885713.

Another associated SNP is rs12885713 of the CALM1 gene, which Zhao et al. found a p-value of 0.034 [24].

### LBX1

The LBX1 gene was identified in three included studies [17, 18, 20]. All three studies identified rs11190870 as a significant SNP. In Takashi et al., no significant statistical association was found; however, in Kou et al. and Liu et al., the association was statistically significant [17, 18].

### MATN1

While two studies both tested for the MATN1 gene, one for the 1p35 marker and the other for rs1149048, both reported no statistically significant association [19, 27].

### CHL1

Moon et al. and Sharma et al. identified rs10510181, an SNP of the CHL1 gene; however, the two studies showed a discrepancy in p-value [12, 19]. While Moon et al. found no association, Sharma and colleagues reported a p-value of 0.021, suggesting an association between this SNP and IS development.

FBN1 and FBN2.

Buchan et al. gave p-value and odds ratio for FBN1 only, FBN2 only, FBN1 or FBN2: the p-value and OR were 0.0041 and 4.2, 0.0307 and 3.0, and 0.00054 and 3.5 respectively [9]. These values all highlight a strong association between the affected gene and the development of scoliosis.

### Quality of Evidence

**Fig. 2:**
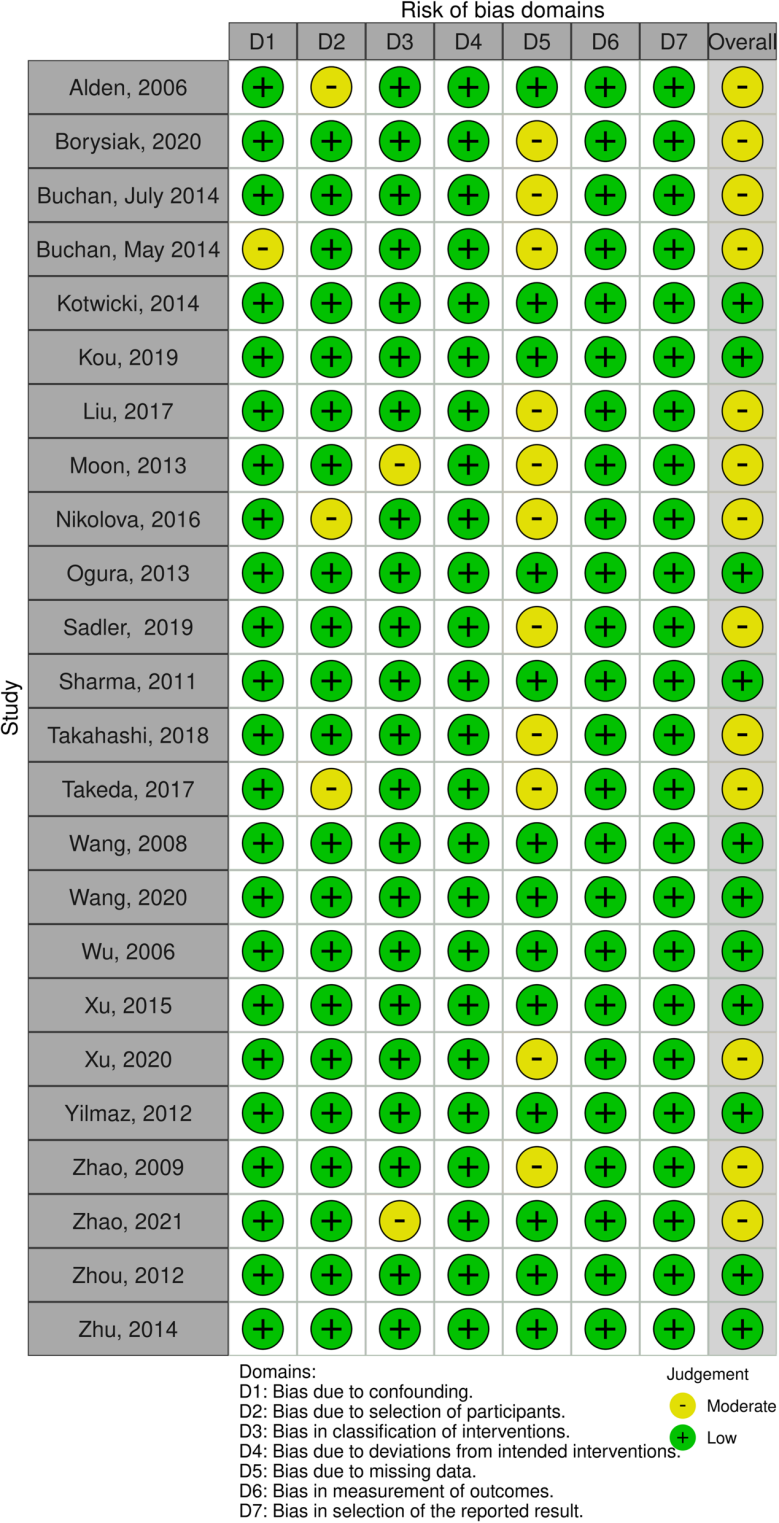
ROBINS-I Diagram

Upon assessment using the ROBINS-I tool, the risk of bias for 11 of the studies was considered “low”, while 13 were found to have a “moderate risk of bias”. “Bias due to missing data” was the most common bias domain, followed by “bias due to selection of participants”. Most of the studies were similar in design and did not precisely describe the enrollment criteria of the participants (Fig. 2).

No Randomized Clinical Trials were not considered eligible; therefore, the RoB-2 tool was not used.

## Discussion

Idiopathic Scoliosis is a multifactorial condition, and the present study focuses on exploring whether specific genetic mutations or polymorphisms could influence its onset [32, 33]. Understanding the genetic basis of this disease may lead to early diagnosis and treatments.

The CALM1 gene, along with CALM2 and CALM3, are genes that code for calmodulin, a calcium receptor protein involved in various cellular processes, including cell differentiation, cell proliferation, and cytoskeletal architecture and function, and metabolic homeostasis [34]. This gene, and more directly calmodulin, has previously been associated with the development of IS and has been shown to play a role in musculoskeletal development [35]. Furthermore, the results showed a positive correlation between a specified SNP of this gene and IS onset [24].

The studies by Buchan [9] and Sadler [7], identified CNV 16p11.2 as having a positive correlation with the onset of scoliosis. The 6p11.2 distal deletion includes the SH2B1 gene involved in leptin and insulin signalling and has been shown to have a polymorphic effect on obesity [7, 36]. More specifically, this gene promotes leptin signalling by stimulating Janus kinases 1 and 2 [36]. A specific study reported the risk of scoliosis as 1.5 times higher in the underweight group compared to both healthy and overweight groups [7, 37]. A study also reported that IS patients had lower leptin levels in serum compared to the control group, a parameter often found in severely underweight patients [37]. This data suggests that there may be involvement of the SH2B1 gene in IS onset thanks to its involvement in leptin signalling, and perhaps its polymorphic effects on weight regulation [7, 36].

Furthermore, data seems to support the idea that distal regions may exert regulatory effects on proximal regions of the CNV, including the TBX6 gene [7]. This is especially significant because TBX6 is related to somite development critical to the axial skeleton [38]. TBX6 compound inheritance has also been shown to lead to congenital vertebral malformations in humans and mice [39], which was the associated pathology reported by Takeda and colleagues [29]. The TBX6 gene was also targeted for testing independently by Takeda et al. and Zhao et al. Unfortunately, these studies did not provide statistical comparisons [4, 29].

Kotwicki, Wang, and Wu et al. looked at estrogen receptors genes, but only the latter two found significant statistical association [16, 28, 30]. These data points reflect the controversial role of estrogen in IS. Estrogen’s role in growth regulation and adaptation has been a target for therapy, especially in adolescents, but these therapies have come with their criticisms [35]. Furthermore, in a study performed by Rusin et al., an asymmetric expression of ESR2 in deep paravertebral muscles was discovered to favour the side of convexity of the spinal curve in IS patients, supporting the idea of a correlation between estrogen and IS [40]. Unfortunately, it is not yet clear whether these findings are causes or consequences of the onset of IS [33].

Three studies focused on the LBX1 gene [17, 18, 20], with two of them finding statistically significant associations with the onset of IS^15,16^. LBX1 mutations have been linked to disruption of paraspinal development, which is regulated by the WNT/beta-catenin pathways [35]. This may be due to its role in muscle embryonic development. LBTX1 gene modulates the migratory routes of hypaxial muscle precursors that are crucial in developing muscle patterns of the limbs [41]. One specific case report showed a microduplication at CNV 10q24.31, only affecting LBX1. This mutation was associated with congenital scoliosis and paravertebral hypotrophy [41]. Microduplication is believed to interfere with migration activity and influence muscle development [41]. Paraspinal muscles play a crucial role in spinal stability and research suggests that muscle-based mechanisms may contribute to IS development [42].

Moon and Sharma identified rs10510181, an SNP of the CHL1 gene [12, 19]. While Moon et al. found no association, Sharma and colleagues suggested an association between this SNP and IS development. CHL1 encodes an axon protein involved in the guidance of thalamocortical axons and the proliferation and differentiation of neural progenitor cells [43]. It has been demonstrated that mutations in this gene disrupt axonal guidance of brain anatomy in mice [43]. Some studies reported that abnormalities in the central nervous system (CNS) could predispose to AIS [43]. The disturbance in the CNS may impair somatosensory function and motor adaptation leading to the asymmetry of the neuromuscular condition [43].

The LBX1 gene, beyond playing a role in embryological muscle development also specifies distinct neuronal subtypes in the spinal cord [42]. LBX1 expression creates a distinction between two neuronal classes generated in the dorsal spinal cord and functions as a selector gene in the fate determination of somatosensory relay neurons [42]. When gait parameters of IS patients were investigated, somatosensory dysfunction showed an impact on dynamic balance control, which may play a role in etiology. Unfortunately, this is another instance where it is unclear whether it is a cause or consequence of IS onset [42].

However, both LBX1 and CHL1 influence the CNS and have both been statistically associated with IS onset.

Data on genetic correlations with IS onset would benefit from some standardizing measures, including more consistent reporting of odds ratio and p-value as statistical measures, a standardized measure for reporting allele frequency, clearer inclusion and exclusion criteria for participants, and more participant data, including sex, age of IS onset, and ethnicity. These measures could improve the quality of preliminary data and allow for a more in-depth and accurate exploration of the genetic correlations with IS onset and facilitate comparison across different studies.

### Limitations

The present review has some limitations. The study did not collect data from randomized control trials and included some low-quality studies.

Secondly, the meta-analysis of results could not be performed due to the heterogeneity of the collected data. Only English-language articles were included, limiting the number of eligible articles. Most of the included studies did not distinguish between early-onset and late-onset scoliosis. This is a limitation because the information on the age of onset may have been relevant in understanding the function of the identified genes, or possibly allowed for discrimination between genes identified in early and late-onset.

Another important point to mention is that due to the complexity of this topic contradicting data was sometimes found when searching for genetic correlations to the onset of IS likely due to its complex and multifactorial nature. The discrepancy between Moon et al. and Sharma et al. results regarding the same gene serves as an example.

Furthermore, the present study does not consider the ethnicity of patients and consequentially the possible genetic differences between ethnic groups in relation to the onset of IS. Although more literature on the subject is required studies have reported differences in the prevalence of IS across various races [44, 45]. For example, a retrospective study by Kebaish et al. found that the prevalence of scoliosis was higher in whites (11.1%) compared to African Americans (6.5%) [44]. However, this parameter was not considered because it was not reported in included studies. The lack of data on ethnicity highlights the need to include this parameter in future studies.

## Conclusions

Several studies show an association between the development of scoliosis and specific genes, SNPs, CNVs and markers. Therefore, identifying genes directly linked to the onset of scoliosis would represent a turning point in the diagnosis and treatment of this condition. However, it is not possible to draw a conclusion, due to the lack of high-quality evidence. For this reason, more numerous and higher-quality studies are needed.

## Data Availability

The datasets generated and/or analysed during the current study are not publicly available due analysis being underway for subsequent publications but are available from the corresponding author on reasonable request.

## Abbreviations

AIS: Adolescent Idiopathic Scoliosis
CS: Case series
CC: Case–Control
CS: Cohort studies
CNV: Copy number variant
EOS: Early onset scoliosis
ESRs: Estrogen Receptor genes
GWAS: Genome wide association studies
IS: Idiopathic Scoliosis
NRCT: Non-Randomized Controlled Trials
PS: Prospective studies
RCT: Randomized Controlled Trials
RS: Retrospective studies
SNPs: Single nucleotide polymorphisms
